# Management of paediatric traumatic brain injury in Sweden: a national cross-sectional survey

**DOI:** 10.1186/s13049-022-01022-4

**Published:** 2022-05-12

**Authors:** Fredrik Wickbom, Linda Persson, Zandra Olivecrona, Johan Undén

**Affiliations:** 1 Department of Operation and Intensive Care, Halland Hospital, Halmstad, Sweden; 2 Lund University, Lund, Sweden; 3 Department of Orthopaedics, Halland Hospital, Halmstad, Sweden; 4 Department of Neurosurgery, Faculty of Health and Medicine, Department for Medical Sciences, Örebro, Sweden

**Keywords:** Head injuries, mTBI, TBI, Children, Guidelines, Initial management, Sweden

## Abstract

**Background:**

Previous studies have shown variations in management routines for children with traumatic brain injury (TBI) in Sweden. It is unknown if this management has changed after the publication of the Scandinavian Neurotrauma Committee guidelines in 2016 (SNC16). Also, knowledge of current practice routines may guide development of an efficient implementation strategy for the guidelines. The aim of this study is therefore to describe current management routines in paediatric TBI on a hospital/organizational level in Sweden. Secondary aims are to analyse differences in management over time, to assess the current dissemination status of the SNC16 guideline and to analyse possible variations between hospitals.

**Methods:**

This is a sequential, cross-sectional, structured survey in five sections, covering initial management routines for paediatric TBI in Sweden. Respondents, with profound knowledge of local management routines and recommendations, were identified for all Swedish hospitals with an emergency department managing children (age 0–17 year) via phone/mail before distribution of the survey. Responses were collected via an on-line survey system during June 2020–March 2021. Data are presented as descriptive statistics and comparisons were made using Fisher exact test, when applicable.

**Results:**

71 of the 76 identified hospitals managed patients with TBI of all ages and 66 responded (response rate 93%). 56 of these managed children and were selected for further analysis. 76% (42/55) of hospitals have an established guideline to aid in clinical decision making. Children with TBI are predominately managed by inexperienced doctors (84%; 47/56), primarily from non-paediatric specialities (75%; 42/56). Most hospitals (75%; 42/56) have the possibility to admit and observe children with TBI of varying degrees and almost all centres have complete access to neuroradiology (96%; 54/56). In larger hospitals, it was more common for nurses to discharge patients without doctor assessment when compared to smaller hospitals (6/9 vs. 9/47; *p* < 0.001). Presence of established guidelines (14/51 vs. 42/55; *p* < 0.001) and written observation routines (16/51 vs. 29/42; *p* < 0.001) in hospitals have increased significantly since 2006.

**Conclusions:**

TBI management routines for children in Sweden still vary, with some differences occurring over time. Use of established guidelines, written observation routines and information for patients/guardians have all improved. These results form a baseline for current management and may also aid in guideline implementation.

**Supplementary Information:**

The online version contains supplementary material available at 10.1186/s13049-022-01022-4.

## Background

Traumatic brain injury (TBI) is recognized as a common cause of death and disability among children worldwide [1]. Most patients (70–98%) are classified as having minimal or mild traumatic brain injury (mTBI) and most will swiftly recover without suffering from any persisting sequelae [2, 3]. However, a minority will suffer life-threatening intracranial complications in need of urgent attention and often rapid surgical intervention [4]. The task of effectively identifying these patients is a clinical challenge.

In 2006, a national survey in Sweden demonstrated differences in management routines for children with TBI [5], where only 27% of hospitals in Sweden had any sort of guideline directing how care should be delivered. Initial assessment in emergency departments (ED) was primarily performed by non-specialists (assistant residents and/or residents) in 96% of cases [5], which could mandate a need for support by senior colleagues or clinical decision aids as seen in adult TBI [6]. A report examining 72 emergency departments from New England, USA, from 2013 showed that 35% of the hospitals did not have clinical practice guidelines for concussion management in adults and children [7].

Lack of established routines may negatively affect patient safety [4]. The use of clinical decision rules (CDR´s) for management of mTBI has been shown to decrease the number of CT scans, without an elevated risk of missing potentially dangerous intracranial haemorrhages [8, 9]. Several clinical decision rules have been published during the last 15 years [4, 10, 11], including a Scandinavian guideline (SNC16) published in 2016 [12].

Introducing a new guideline can be difficult [13]. A guideline adapted and applicable to the clinical setting [14], and a tailored dissemination process, with knowledge about cultural factors and attitudes to guideline use [15], is important for successful implementation and guideline adherence [6, 16].

As part of a series of studies aimed to increase understanding, facilitate and tailor implementation of the SNC16 guideline, this study aims to describe current management routines in paediatric TBI on a hospital/organizational level in Sweden. Secondary aims are to analyse differences over time, when compared to a study conducted with a similar method from 2006 [5], assess the current dissemination status for the SNC16 guideline and to analyse possible management variations between hospitals.

## Methods

### Study design

This is a sequential, observational, cross-sectional survey concerning the initial (first 24 h) management routines in paediatric TBI in Sweden, including an analysis of evolvement in routines since 2006. STROBE guidelines for reporting cross-sectional studies were used (Additional file 1).

### Setting

All hospitals with an emergency department (ED) in Sweden, with capacity to assess and manage children suffering from TBI, were included. Hospitals lacking an emergency department or those only managing adults were excluded.

### Participants

The head of the department for general surgery or emergency medicine in each hospital was often initially contacted, recommending a suitable respondent in their organization. This person was subsequently contacted by phone or e-mail before the survey was emitted by e-mail. A respondent was regarded as suitable if him/her was in a position mandating overview of paediatric TBI routines in the organization, for example as author of local routines or an operations manager in the ED. The survey was answered once per participating hospital.

### Variables

Data was collected using a web-based system (EsMaker®, Entergate AB). The survey was open from June 2020 to March 2021. Reminders were sent by e-mail to non-responders until > 90% response rate was reached. Neither personal data nor individual patient records were collected, but rather general information on management recommendations/routines in the hospital.

### Data sources/measurement

The questionnaire was designed in collaboration with members of the Scandinavian Neurotrauma Committee (SNC), to be comparable with data from a similar study from 2006 [5], as described below. The survey was structured into five different sections. Table 1 exemplifies questions from each section. A pilot version was sent to six hospitals and after minor adjustment, the complete form was dispersed.

**Table 1:** The questionnaire – sections and exemplified main questions

*Section 1: Background information*
Name of hospital
Presence of written guidelines guiding initial management of children within 24 h of TBI in the organization?
* Section 2: Initial treatment in the emergency department*
What clinic is responsible for paediatric patients suffering from TBI?
Are these patients cared for by specialists or non-specialists?
*Section 3: Radiology*
What primary radiology modality is recommended?
Access to anaesthesiologist and diagnostic radiology
*Section 4: In-hospital observation*
What department are patients in need of in-hospital observation admitted to?
What parameters are recommended to be monitored during hospitalization?
*Section 5: Discharge and follow-up*
Are patients and guardians per routine provided with discharge information?
Does your hospital arrange follow up?

To analyse changes in recommendations over time, a comparison with data from 2006 was done. In this report by Åstrand et al. [5], investigators aimed to describe current management practice for children within mTBI in Sweden by distributing a questionnaire by mail to 51 hospitals with emergency departments in Sweden.

### Bias

To reduce reporting bias, we made sure that the respondents were fully aware of the local routines.

### Study size

We aimed for a response rate of at least 90%, ensuring an adequate representation of the Swedish health care for these patients.

### Quantitative variables

Cross-comparison was performed between four categories depending on the size of the hospital: local, regional, university and children´s hospital. Due to known small numbers in the last two groups, the categories were a priori dichotomised to smaller hospitals (local and regional) and larger hospitals (university and children´s hospitals).

In analyses regarding level of experience of the responsible clinician (Table 3), manging speciality in Table 4 (paediatric vs. non-paediatric) and Table 5 (neurology vs. non-neurology), categorization has been done as presented below.

Table 3 presents of a rating of what experience level doctors in ED possess. Categories are “assistant physician, dependent”, “assistant physician, independent”, “intern”, “resident” and “specialist”. For each experience level the respondent rated on a 5-grade scale (always; often; sometimes; rarely; never) how frequently this category is involved in TBI management. A dichotomisation of the experience levels to “specialist” and “non-specialist level” (in which categories: “assistant physician, dependent”, “assistant physician, independent”, “intern” and “resident” were merged) was done for the analysis. To further simplify presentation, grade “always” and “often” was merged (implying the “most common” experience level for clinicians manging children with TBI, presented for respective hospital size. This means that grade “sometimes”, “rarely” or “never” won´t be presented in the table. Merging of experience levels and response options means that the aggregated total response rate will not be 100%. Percentages are calculated as number of responses per total hospitals in each category.

In Table 4 managing clinic (speciality of the clinic) is categorized as paediatric (paediatric surgery; paediatrics; paediatric neurology; paediatric orthopaedics), non-paediatric (neurology; general surgery; internal medicine; orthopaedics or another speciality) or emergency medicine. Table 5 is presented in the same way, with categorization based on neurology speciality (paediatrics; paediatric neurology; neurology; internal medicine), non-neurology (paediatric and general surgery; orthopaedics) or emergency medicine. For each type of clinic, respondent was asked to rate, in analogous way, on a 5-grade scale (always; often; sometimes; rarely; never) how frequent this speciality manages children with TBI at their emergency department. With the same methodology as in Table 3, grade “always” and “often” was merged (implying the “most common” managing clinic/speciality. As above, presentation is based on hospital size.

### Statistical methods

Data was summarized and presented using descriptive statistics. Differences over time were analysed comparing data from Åstrand et al. [5] with the present data. Statistical analysis was performed using Fisher's exact test to detect differences between groups, when indicated. A two-tailed *p*-value of < 0.05 was considered significant. As answers for a certain question were not always 100% complete, the total number of responses is given with each question.

An ethical advisory opinion was granted by the Swedish Ethical Review Authority, Dnr 2020–02,693.

## Results

### Characteristics of the hospitals

**Fig. 1:**
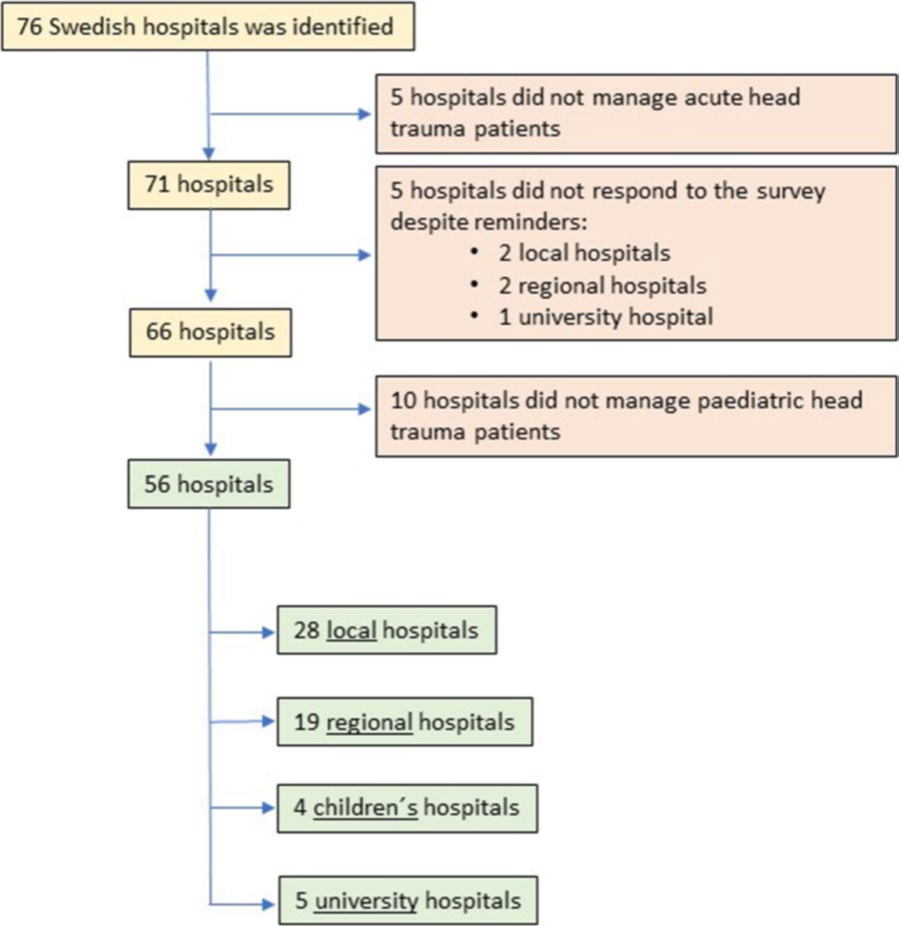
Flowchart—participating hospitals

We identified 76 hospitals in Sweden; 5 of these did not manage TBI at all. Of the remaining 71 hospitals, responses were returned from 66 (overall response rate 93%). 56 of these managed paediatric patients and one respondent in each centre received the study questionnaire. Size and type of included and excluded hospitals are described in Fig. 1.

Nine (16%) hospitals had a transfer time of > 2 h to neurosurgical service. 96% of hospitals (54/56) reported 24 h accessibility to a CT scan and all had access to around-the-clock anaesthesiology support.

### Management recommendations

Most of the hospitals (76%; 42/55) used an established guideline to aid in emergency management of children with TBI (Table 2). The most used guideline was the SNC16 guideline [12], in part or fully used by 31 hospitals (55%). Following this, the most described was a local modification of pre-existing validated guidelines (such as PECARN [4]) or local guidelines based on local expert opinion.

**Table 2:** Use of guideline for management of paediatric TBI

	Local hospital *n* (%)	Regional hospital *n* (%)	Children´s hospital *n* (%)	University hospital *n* (%)	Total *n* (%)
Established guideline	20 (71)	13 (68)	4 (100)	5 (100)	42 (76)
No guideline	7 (25)	4 (21)	0	0	11 (20)
Unknown	0	2 (11)	0	0	2 (4)
Total	27 (96)	19 (100)	4 (100)	5 (100)	55 (98)

Numbers presented for respective hospital category and all hospital categories in total. One respondent (*n* = 1) from a local hospital did not respond to this question (response rate 55/56, 98%)

### Emergency department routines

Patients were predominantly assessed by non-specialists (84%; 47/56; Table 3) and, apart from EDs in children's hospitals, rarely seen by a doctor from a paediatric speciality (13%; 7/56; Table 4). Most commonly, the assessing doctor was from the department of general surgery (71%; *n* = 40). In 19 hospitals (34%), emergency medicine physicians often or always performed the first assessment, and it was uncommon (5%) to meet a doctor from a neurological speciality (Table 5).

**Table 3:** Level of experience of responsible clinician

	Local hospital *n* (%)	Regional hospital *n* (%)	Children´s hospital *n* (%)	University hospital *n* (%)	Total *n* (%)
Non-specialist *“assistant physician, dependent”,* *“assistant physician, independent”,* *“intern” and “resident” are merged*	22 (79)	18 (95)	3 (75)	4 (80)	47 (84)
Specialist	10 (36)	3 (16)	3 (75)	3 (60)	19 (34)

*Example* There was in total 22 responses in the non-specialist category deriving from local hospitals, implying that in 22 of the 28 local hospitals (79%) it is common (“often” or “always”) that non-specialists are managing children with TBI

**Table 4:** Responsible clinic: Paediatric versus non-paediatric specialities

	Local hospital *n* (%)	Regional hospital *n* (%)	Children´s hospital *n* (%)	University hospital *n* (%)	Total *n* (%)
Paediatric speciality	0	1 (5)	4 (100)	2 (40)	7 (13)
Non-paediatric speciality	22 (79)	18 (95)	0	2 (40)	42 (75)
Emergency medicine	10 (36)	6 (32)	0	3 (60)	19 (34)

*Example* There was in total 10 responses in the emergency medicine category deriving from local hospitals, implying that in 10 of the 28 local hospitals (36%) it is common (“often” or “always”) that emergency medicine physicians are managing children with TBI

**Table 5:** Responsible clinic: neurology versus non-neurology specialities

	Local hospital *n* (%)	Regional hospital *n* (%)	Children´s hospital *n* (%)	University hospital *n* (%)	Total *n* (%)
Neurology speciality	0	0	1 (25)	2 (40)	3 (5)
Emergency medicine	10 (36)	6 (32)	0	3 (60)	19 (34)
Non-neurology speciality	20 (71)	18 (95)	3 (75)	2 (40)	43 (77)

*Example* There was in total 20 responses in the non-neurology category deriving from local hospitals, implying that in 20 of the 28 local hospitals (71%) it is common (“often” or “always”) that non-neurology physicians are managing children with TBI

15 hospitals (27%) reported that patients were occasionally discharged by a nurse at triage without any doctor assessment. 8 of these units had written guidelines concerning this procedure; 6 of these used the SNC16 guidelines. Almost all hospitals (*n* = 54) use CT as the primary choice of radiology modality to exclude intracranial complications.

### In-hospital observation

The possibility for in-hospital observation was reported for 75% of the hospitals (42/56), most commonly (64%) in a general ward. In 46% (13/28) of the local hospitals, external transfer to another hospital was mandated if the patient had to be admitted for any reason (Table 6). In local hospitals, 50% (7/14) of children were observed in a non-paediatric ward. In larger hospitals, most children were admitted to paediatric wards (96%; 25/26). In one hospital, the Intensive Care Unit (ICU) was used for observation.

**Table 6:** Possibility of in-hospital observation

	Local hospital *n* (%)	Regional hospital *n* (%)	Children´s hospital *n* (%)	University hospital *n* (%)	Total *n* (%)
Possibility of in-hospital observation	15 (54)	18 (95)	4 (100)	5 (100)	42 (75)
No possibility of in-hospital observation	13 (46)	1 (5)	0	0	14 (25)

Numbers presented for respective hospital category and all hospital categories in total

**Fig. 2:**
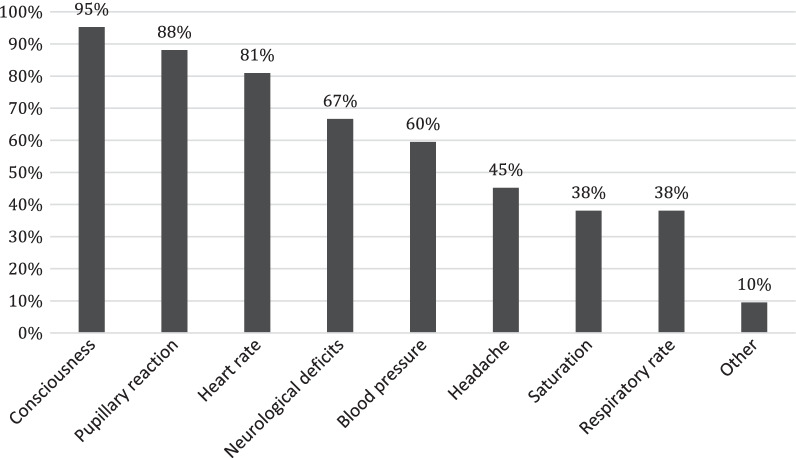
Parameters evaluated during in-hospital observation due to paediatric TBI. Respondents could choose more than one alternative (*n*_tot_ = 42)

During the observation period, level of consciousness was the parameter most often evaluated (95%), followed by pupillary reaction, heart rate and neurological deficits (Fig. 2).

**Fig. 3:**
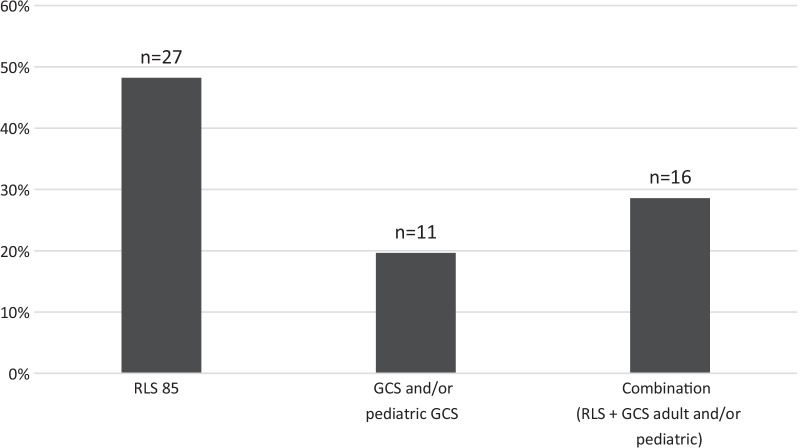
Scale used for assessment of level of consciousness. Respondents were asked to report which scale(s) that was used at their hospital for assessment of level of consciousness. More than one alternative could be marked. RLS85 [17], GCS (adult version) and GCS (paediatric version) was prespecified options. Three (*n* = 3) respondents reported use of AVPU-scale (Alert, Verbal, Pain, Unresponsive) in addition to GCS or RLS85

When evaluating level of consciousness, RLS85 [17] was the most frequently reported answer, either alone (48%) or in combination with other assessment scales (29%) (Fig. 3).

97% of hospitals with capacity for in-hospital observation (41/42) reported their observation routines. In 46% (19/41), elements of the SNC 16 routines were used regarding type, frequency and/or duration of observation and in 32% (13/41) the SNC16 was the sole guiding routine for in-hospital management. 44% (18/41) of the hospitals allowed individual doctor prescription of observation criteria, and in 27% (11/41) no other routines other than doctor prescribed observation was used.

Routines for CT scanning in admitted children were mostly (solely or in combination with other written routines) based upon doctor discretion (65%, 27/41). In 49% (20/41) of the hospitals a written routine in some form guided CT scanning in admitted children, mainly the SNC16 (31%, 13/41). 56% (23/41) of hospitals reported lack of discharge-criteria following observation. Concerning information to patients/guardians at discharge, 15% (8/55) provided only written information, 9% provided only oral information and 71% provided both, with only 5% not providing discharge information at all.

### Follow-up

38% (21/56) of all hospitals could arrange a follow up assessment if needed, which was relatively more common at large hospitals (7/9, 78% vs. 14/47, 30%) and usually either at a paediatric outpatient clinic (52%) and/or in the primary care sector (48%). 46% (26/56) did not provide or plan follow-up in children following TBI, this was more common in small hospitals (25/26). There was a significant difference in follow-up routines between small (local and regional) and larger (university and children´s) hospitals (*p* = 0.015).

### Other analyses

A significantly higher presence of physicians in paediatric specialities (*p* < 0.001) was reported for emergency departments in larger hospitals. No difference in presence of specialists (*p* = 0.17) or presence of established guidelines in hospitals emitted recommendations (*p* = 0.18) was found compared to smaller hospitals.

Nurse triage without doctor involvement was more common in large hospitals (*n* = 6/9) compared to small ones (*n* = 9/47) (*p* < 0.001).

### Changes in management routines

In 2006 survey, 27% (14/51) of the hospitals had written management criteria/routines. A significant increase (76%; 42/55) was observed over time (*p* < 0.001). It is more uncommon to use ICU for observation today (1 vs. 10 hospitals, *p* = 0.020). The presence of written observation routines (defined as presence of a local written routine regarding in-hospital observation and/or use of the SNC16 recommendation for in-hospital observation) is more common today when compared to 2006 (69% compared to 31%, *p* < 0.001). Finally, the possibility of follow-up after discharge did not differ between 2006 and the current study (*p* = 0.22). See Table 7 for details.

**Table 7:** TBI management in Swedish hospitals, comparison between 2006 [14] and the present study

	2006 survey *Åstrand *et al. *n* (%)	Current survey *n* (%)	*p*-value
Using established guidelines	14/51 (27%)	42/55 (76%)	*p* < *0.001*
ICU as observation unit	10/51 (20%)	1/42 (2%)	*p* = *0.02*
Written observation routines	16/51 (31%)	29/42 (69%)	*p* < *0.001*
Possibility of follow-up after discharge	13/51 (25%)	21/56 (38%)	*p* = 0.22

## Discussion

This study describes the current management routines of children with TBI in Sweden. Most hospitals (76%) use an established guideline to aid in management, which is in line with results from the US (65%), in a study by Stern et al. [7]. SNC 16 was the most-used guideline, despite the lack of formal national implementation. This may reflect an unwillingness to use other decision rules based on high quality evidence, such as the PECARN rule [4], which may increase CT use [18, 19]. The SNC16 guideline (concerning mild and moderate TBI) [12], together with the Brain Trauma Foundation guidelines covering severe TBI [20], were reported as the paediatric guidelines with the highest overall quality in a recent systematic review and quality analysis [21], receiving 45/49 and 44/49 points, respectively. Before formal implementation however, the SNC16 should be adequately validated in the Swedish health care setting, a process which is currently underway. Considering differences over time, a significant increase in use of established guidelines has occurred since 2006 (76% vs. 27%). This result is in itself of importance, as the use of evidence-based guidelines have been shown to improve outcome in TBI [22].

In 27% of the hospitals, in particular larger hospitals (*p* < 0,001), we found that nurses can discharge from the ED without doctor assessment. In most of the hospitals (8/15), these patients are discharged using a guideline, mainly the SNC16. This type of management may be safe and efficient, despite not officially being supported by the SNC16 guideline, and therefore needs further investigation.

Despite using similar risk-assessment strategies, the logistics involved with more remote or smaller hospitals may impact patient management. 16% of the hospitals reported having at least 2 h transfer time to the nearest neurosurgical unit. Also, approximately half (13/28, 46%) of the smaller local hospitals could not offer in-hospital observation. These aspects of non-patient related factors may influence decision-making, reflecting real-world issues in Sweden and is not specifically addressed in the SNC16 guideline, nor in others [4, 10, 11], and warrant further investigation.

More than half of hospitals did not have specific discharge criteria. Although evidence is lacking in this area, written discharge criteria may facilitate management and promote equality in patient management. Discharge from the hospital should be accompanied with information regarding the injury, what to expect and when to seek health care. Most hospitals provided this with only 3 hospitals (5%) stating that they do not per routine provide such information.

This study has several limitations. Data on individual patient management is lacking in this study, as focus has been to describe current routines, practice variation and evolvement in management recommendations between hospitals and over time. As patient management does not necessarily adhere to recommendations emitted by hospitals and medical organs, results must be interpreted with caution. Also, data in this study refers to Swedish health care, and the results may therefore not be applicable to other countries. There is also a risk for reporting bias as information gathered from a single respondent may not always accurately reflect clinical practice, despite efforts to ensure that the respondent was fully aware of all aspects of TBI management in their hospital organization. Also, the completeness of data was not always 100%, which may also account for some errors. However, these issues were limited and assessed to have negligible effect on the overall results. Percentage of missing data are reported for each question.

The strengths of this study lie in the high response rate and the on-line survey system which increases response accuracy and minimises ambiguous answers. As this group did a similar survey in 2006 [5], most questions are similar which allows a reliable comparison over time.

Although this study shows an increasing use of guidelines over time, many hospitals still seem to use management recommendations based upon weaker scientific methods or routines based upon local expert opinion. As a majority of these children will be managed by inexperienced doctors from varying specialities, especially in smaller hospitals, the findings support the need of a nationally implemented and accepted guideline to aid in decision making.

Following successful validation, optimal adherence to a guideline is desired. There is a need for development of a structured dissemination and implementation process in Sweden. However, further investigation of how intended users of the guideline assess its user-friendliness as well as facilitators and barriers for guideline adherence is warranted, as data on this is lacking in this study. Data on individual patient management in Sweden is also needed.

## Conclusions

Hospital management routines for children with TBI varies in Sweden, with some differences occurring over time. Use of established guidelines, written observation routines and information for patients/guardians have all improved. These results will together with upcoming studies form a baseline for the process to design a tailored dissemination and implementation plan for the SNC16 guideline in Sweden.

## Supplementary Information

**Additional file 1. **STROBEStatement—Checklist of items that should be included in reports ofcross-sectional studies.

## Data Availability

The datasets used and/or analysed during the current study are available from the corresponding author on reasonable request.

## References

[1] Araki T, Yokota H, Morita A (2017). Pediatric traumatic brain injury: characteristic features, diagnosis, and management. Neurol Med Chir.

[2] Babcock L, Byczkowski T, Wade SL, Ho M, Mookerjee S, Bazarian JJ (2013). Predicting postconcussion syndrome after mild traumatic brain injury in children and adolescents who present to the emergency department. JAMA Pediatr.

[3] Barlow M, Schlabach D, Peiffer J, Cook C (2011). Differences in change scores and the predictive validity of three commonly used measures following concussion in the middle school and high school aged population. Int J Sports Phys Ther.

[4] Kuppermann N, Holmes JF, Dayan PS, Hoyle JD, Atabaki SM, Holubkov R (2009). Identification of children at very low risk of clinically-important brain injuries after head trauma: a prospective cohort study. The Lancet.

[5] Astrand R, Unden J, Bellner J, Romner B (2006). Survey of the management of children with minor head injuries in Sweden. Acta Neurol Scand.

[6] Tavender EJ, Bosch M, Gruen RL, Green SE, Knott J, Francis JJ (2014). Understanding practice: the factors that influence management of mild traumatic brain injury in the emergency department-a qualitative study using the theoretical domains framework. Implement Sci.

[7] Stern RA, Seichepine D, Tschoe C, Fritts NG, Alosco ML, Berkowitz O (2017). Concussion care practices and utilization of evidence-based guidelines in the evaluation and management of concussion: a survey of new England emergency departments. J Neurotrauma.

[8] Brenner D, Elliston C, Hall E, Berdon W (2001). Estimated risks of radiation-induced fatal cancer from pediatric CT. AJR Am J Roentgenol.

[9] Elmoheen A, Salem W, Bashir K (2021). Reducing unnecessary CT scan of the head for minor paediatric head injuries at the emergency department. BMJ Open Qual.

[10] Dunning J, Daly JP, Lomas J, Lecky F, Batchelor J, Mackway-Jones K (2006). Derivation of the children's head injury algorithm for the prediction of important clinical events decision rule for head injury in children. Arch Dis Child.

[11] Osmond MH, Klassen TP, Wells GA, Correll R, Jarvis A, Joubert G (2010). CATCH: a clinical decision rule for the use of computed tomography in children with minor head injury. Can Med Assoc J.

[12] Astrand R, Rosenlund C, Unden J (2016). Scandinavian guidelines for initial management of minor and moderate head trauma in children. BMC Med.

[13] Vedin T, Edelhamre M, Karlsson M, Bergenheim M, Larsson PA (2017). Management of traumatic brain injury in the emergency department: guideline adherence and patient safety. Qual Manag Health Care.

[14] Donnell Z, Hoffman R, Myers G, Sarmiento K (2018). Seeking to improve care for young patients: development of tools to support the implementation of the CDC Pediatric mTBI Guideline. J Saf Res.

[15] Derbyshire S, Maskill V, Snell DL (2021). Do concussion clinicians use clinical practice guidelines?. Null.

[16] Brolliar SM, Moore M, Thompson HJ, Whiteside LK, Mink RB, Wainwright MS (2016). A qualitative study exploring factors associated with provider adherence to severe pediatric traumatic brain injury guidelines. J Neurotrauma.

[17] Starmark JE, Stålhammar D, Holmgren E (1988). The reaction level scale (RLS85) Manual and guidelines. Acta Neurochir (Wien).

[18] Undén J, Dalziel SR, Borland ML, Phillips N, Kochar A, Lyttle MD (2018). External validation of the Scandinavian guidelines for management of minimal, mild and moderate head injuries in children. BMC Med.

[19] Babl F, Borland M, Phillips N, Kochar A, Dalton S, McCaskill M (2017). Accuracy of PECARN CATCH, and CHALICE head injury decision rules in children: a prospective cohort study. Lancet.

[20] Kochanek PM, Chair V, Carney N, Adelson PD, Ashwal S, Bell MJ (2012). Guidelines for the acute medical management of severe traumatic brain injury in infants, children, and adolescents-second edition author affiliations project management external peer reviewers. Pediatr Crit Care Med.

[21] Appenteng R, Nelp T, Abdelgadir J, Weledji N, Haglund M, Smith E (2018). A systematic review and quality analysis of pediatric traumatic brain injury clinical practice guidelines. PLoS ONE.

[22] Vavilala MS, Kernic MA, Wang J, Kannan N, Mink RB, Wainwright MS (2014). Acute care clinical indicators associated with discharge outcomes in children with severe traumatic brain injury*. Crit Care Med.

